# Insight, Motivation and Outcome in Drug Treatment for Offenders: A Review of the Recent Literature

**DOI:** 10.4172/2155-6105.1000210

**Published:** 2015-01-10

**Authors:** Rebecca Linn-Walton, Tina Maschi

**Affiliations:** Fordham University Graduate School of Social Service, New York 10023, United States

## Abstract

Researchers in addiction and psychotherapy have long agreed that insight into problem severity and motivation for treatment are important client factors in successful treatment. For offenders these factors are linked to recidivism and relapse rates post-treatment. Authors in both fields agree that the combination of insight and motivation are key to positive treatment outcomes. However, this literature review found little effort to measure these factors in substance abuse literature with offenders. Articles identified contained the terms ‘motivation;’ ‘insight;’ and ‘drug treatment’ were paired with the term ‘offenders’ in varying combinations to identify articles meeting study criteria. Inductive analysis revealed that the majority of the articles did not measure insight and motivation, nor did they measure outcomes. Only seven of the 16 articles included measures of insight and motivation. Of these, only one study measured outcome as well. In addition, qualitative aspects of insight and motivation were not accounted for by assessments used. Recommendations for future research include measuring insight and motivation as well as treatment outcome, and tailoring treatment for this population accordingly, so as to better predict recidivism rates post-treatment.

## Introduction

### Background

Consequences for abusers of illegal drugs are severe and often include involvement in the criminal justice system. The most recent report from the United States Bureau of Justice reported that in 2009, 33% of the cases tried criminal courts in the United States were for drug charges [1]. While this statistic alone is sizable, it does not account for the significant amount of crimes committed by individuals with histories of drug use and abuse [2–4]. While many scholars agree that the population of offenders with drug problems is notable, actual statistics vary, and those cited are often not current. Trends suggest that the number of arrests for drug-related charges is increasing, and that as much as 50% of those convicted of a crime were under the influence of a substance at the time of arrest [5,6]. These findings indicate that many individuals are in the prison system for various crimes, yet can have underlying drug problems that cause them to offend.

These statistics show that drug charges are treated as serious offenses, and that a drug-related arrest almost always leads to sentencing, whether that sentence is probation, imprisonment, or mandated treatment [1]. In addition, while Motivans reported that the average federal court conviction rate was 78%, the rate of conviction for drug-related charges was 91%. These sentencing practices suggest that drug abusers are being treated in a punitive manner, which can have negative effects on their willingness to engage in treatment, and outcomes such as recidivism and continued abuse of illegal substances [7]. In addition, individuals with drug problems have high rates of recidivism [2].

This last fact has had the positive impact, however, of leading to the majority of individuals sentenced for drug-related charges being given substance abuse treatment as part of sentencing [3,8,9]. This atmosphere in turn lowers patient insight into their responsibility for their problems, and motivation for treatment. Promising practices include court-mandated community and outpatient drug treatment programs, both in and out of the drug court system, as well as drug treatment programs inside prisons.

### Motivation

Evidence suggests that insight and motivation have been cited as important factors in treatment [10–14]. There is a large body of literature about the importance of measuring motivation with substance abuse populations [16,10–12,17]. These authors agree that for offenders, measurement of motivation for offenders is even more important, as it predicts likelihood of relapse and recidivism. Knight et al. [12] developed the Texas Christian University Criminal Justice Client Evaluation of Self in Treatment (TCU CJ CEST). This study uses the definition of motivation contained in the TCU CJ CEST, which is a series of instruments testing levels of engagement, desire to engage in, readiness, and commitment to treatment.

### Insight

The body of literature on insight for offenders in treatment is much more limited than for motivation. There is a large body of literature on this topic in psychotherapy [11,14,17]. For this particular population-offenders-insight refers to personal accountability in the form of the ability to understand one’s role in having been incarcerated [10]. Motivation is defined as the participant’s overall commitment to engaging in and completing treatment [18]. However, this research is plagued with definitional issues since these studies differ in implicit definitions of insight and motivation, and some studies do not actively define the terms at all. In addition, the largest contribution to this issue is psychotherapy literature, which does not include samples of substance abusing offenders (Linn-Walton, unpublished manuscript). For the purpose of this paper, insight is defined as: recognition of problem severity; desire for help; and personal commitment to treatment [7,19].

## Measurement and Outcome

While there is a large body of literature examining drug treatment and recidivism, as well as program evaluations, there is little information available about offender characteristics and their role in insight and motivation and their effects on treatment outcome. Evidence has identified that treatment adherence and cessation of substance abuse is an important factor in lowering relapse and recidivism [7]. In contrast, Roberts et al. [16] has identified a positive correlation between individualized treatment plans and treatment outcome. This finding indicates that treatment aimed at improving individual participant characteristics such as insight and motivation has a positive effect on behaviour post-treatment. However, the link between individualized plans and the individual who receives this treatment has been under-evaluated. The current review will identify and examine the factors of motivation and insight, and their role in drug treatment for individuals with histories of incarceration as identified in the existing literature. Specifically, a content analysis was conducted for articles on this topic, to identify the role of insight and motivation on treatment adherence, participation, engagement, and the outcomes of reduced drug abuse and recidivism rates. This study aimed to evaluate the existing literature for conceptualization and measurement of insight and motivation for offenders in treatment, and how these factors affected outcomes of relapse and recidivism.

## Methods

The specific research questions to guide analysis and be answered are: 1) how are the terms motivation and insight defined and operationalized in this area of research? 2) (How) are these terms integrated into treatment methods and approaches? 3) (How) are these terms integrated into outcome literature? 4) How does research suggesting to strengthening these factors in clients and treatment? 5) What role does motivation and insight play in client experience during treatment? 6) How are insight and motivation important to treatment for this population in particular?

## Sample Identification

### Steps of Content Analysis

The content analysis was conducted in three stages: 1) article identification; 2) article review; and 3) analysis of the data collected. Articles were searched and selected between the spring of 2012 and the end of 2014. Step one involved identifying those articles that met selection criteria. The second step involved analysing and coding the articles selected, and the final step was to write up this report on the findings. A comprehensive review of the available literature was conducted in order to identify articles that examined the role of insight and motivation in treatment outcome. Articles English language, in peer-reviewed journals located through EBSCOhost, which includes 59 databases with journals on the topic of drug treatment for offenders, including Psych ARTICLES, Medline, and soc INDEX. These databases included a wide variety of research on drug treatment, insight, motivation, outcome, and criminal offenders undergoing treatment. Key words used were: “insight;” “motivation;” “incarceration;” “offenders;” and “drug treatment,” used in different combinations. These terms were chosen based on terms used in the literature review. Finally, additional articles meeting criteria for inclusion were located through the reference sections of the initial articles located.

### Inclusion and Exclusion Criteria

Inclusion and exclusion criteria were used to determine the sample. A total of 92 articles were located. Articles were selected for inclusion in the sample only if they: (1) were published after 2000; (2) included a sample of adult offenders; and (3) included a sample of offenders from the United States. Only articles written after 2000 were used, as evidence suggests drug treatment programs are rapidly changing in both their orientations and interventions used [16]. Articles chosen for the sample included samples containing adult offenders, as adolescents are developmentally different from adults. In addition, as treatment needs differ for adolescents and adults in treatment, only articles using adults were included in the final sample. While there were several articles on the topic of insight and motivation for offenders in drug treatment in programs outside the US, these articles were not included, as sentencing practices and available treatments for each country is different [5]. Only English language articles were located, so there was no issue of needing to limit or translate articles. Out of these articles came the 16-article sample this study examined.

### Analysis

The content analysis was used both quantitative and qualitative analysis. Frequencies of methods and characteristics across the studies were recorded and analysed. The qualitative analysis utilized in-vivo content analysis coding and thematic coding techniques, as outlined by Berg and Braun and Clarke [20,21]. Berg [20] identifies this method as helpful in identifying both implicit and explicit content on the levels of key words or phrases, paragraphs, and overall themes of the studies [21].

While Berg identifies this process as informed by grounded theory qualitative research; Braun and Clarke [21] suggest that in areas that have little available research, grounded theory practices should not inhibit coding procedures. During the analysis portion of this study, the author attempted to allow the data and background knowledge to inform the coding choices as often as possible. Knowledge in the areas of drug treatment, incarceration, and factors of effective clinical treatment was essential to shaping appropriate and meaningful codes and thematic findings. This form of inductive analysis allowed the data to shape the analysis Braun and Clarke [21] systematize Berg’s previous work, describing a 6-stage coding process: 1) familiarizing oneself with the data through numerous readings; 2) generating initial codes; 3) searching for themes; 4) reviewing and editing the codes; 5) defining and naming themes; and 6) producing the report (Braun and Clarke, p. 87). Both Berg [20] and Braun and Clarke [21] state that this process is especially conducive to analysing data in an under-researched field, as it allows the researcher to approach the data without preconceived theories or codes, and allows for flexible coding and thematic identification. The author employed these methods to evaluate the sample.

## Results

### Quantitative

The 16 articles included for review were published between 2000 and 2014. Table 1 includes quantitative findings of the sample. Seven of the studies published in interdisciplinary journals on the topic of the criminal justice system, two published in journals on drug abuse issues, and one article was published in a social work journal. Criminal Justice and Behaviour was the most widely published journal (n=4). Most studies included quantitative methods (n = 9), six cross-sectional designs, and seven employed a longitudinal design.

Sampling strategies were largely non-probability sampling. Only one study employed random sampling techniques. Additionally, sample size varied greatly across the studies, from 20, to 3,266. However, most of the studies evaluated (n=8) had samples larger than 200. Of the 16 studies in the sample, seven included statistics on participants’ age. Nine studies reported a sample that varied in terms of race/ethnicity. Half of the studies (n=8) used participants from several states in the US, though all of the samples were drawn from Southern United States, the Southwest, and California. Respondent rates were not available.

There were inconsistencies in the use of conceptual or nominal definitions for key variables, which included the use of no definition and/or the use of implicit or explicit definitions across the study sample. Four studies did not explicitly define motivation or insight, and six studies used and implicit definition of insight. Explicit definitions of insight, motivation, and desired treatment outcomes were operationalized in the measures chosen for the sample studies (Table 1).

## Qualitative

### Thematic findings

Table 2 refers to the thematic codes used when evaluating the sample. Inductive analysis found that insight was not thematically limited to individual awareness of problem severity. Instead, studies analysing treatment pointed to the treatment model itself recognizing problem severity. This finding was true of motivation as well. Themes arose indicating that there is both individual motivation level as well as treatment designed to encourage motivation. Bui and Morash [2] found that family and support networks play a positive role in motivation as well and that this was positively associated with treatment outcomes of reduced recidivism or relapse.

Individual level insight was defined as awareness of having a drug problem, acknowledging the severity of the problem, and awareness that drug-related criminal activity was responsible for one’s criminal history, rather than outside individuals/circumstances [6,7,10,12,15,16,18]. The CJ CEST was able to operationalize individual insight as level of criminal thinking in order to measure participant levels of insight. Though Melnick et al. [7] did not employ the CJ CEST the study did seek to measure insight in a similar manner, measuring awareness of problem against problem severity to assess for insight. These seven articles that measured insight found that higher individual levels of insight was a pathway to stronger treatment adherence and participation, and even improved counseling rapport. In the articles that did not explicitly define insight, it was unclear how insight differed from motivation in creating better outcomes for participants, such as the studies by Bui and Morash, Kubiak, and Shaffer, Hartman, and Listwan [2,13,22]. However, these articles offered aspects of insight not discussed elsewhere. Kubiak’s study was the only study to mention that diagnoses other than drug addiction can affect individual levels of insight [13]. Bui and Morash cited individual insight into the need for social networks to reduce post-release/treatment recidivism was an important factor in positive treatment outcome [2]. Shaffer, Hartman, and Listwan found that the punitive nature of the court system for offenders with drug abuse problems often lowers levels of insight in participants [22].

Individual levels and characteristics of motivation were defined, measured, and accounted for in treatment discussions much more clearly than were insight levels and definitions. All eight studies to employ the CJ CEST defined motivation as a combination of readiness for treatment, treatment engagement, and motivation to attend treatment. Hiller, et al. [18] found that higher levels of insight created increased motivation for participants. Bui and Morash [2] did not explicitly define motivation, but cited a desire for change as a major component of positive behavioural changes upon release. No matter what language they used to describe motivation, all 16 studies found that individuals with higher levels of motivation to attend, engage, and complete treatment did better during and after treatment.

In addition to defining and measuring individual motivation, several articles found a difference between internal and external motivation to be an important factor in the pathway to positive treatment outcomes. Bui and Morash [2] found that the presence of positive support networks contributed to increased motivation to succeed in treatment, while negative outside influences lowered levels of personal motivation. The seven studies that used the CJ CEST conceptualized negative positive outside influence as participant belief that they had been incarcerated and sent to treatment not as a result of their drug use and drug-related criminal activity, but because the court system was against them, this was a result solely of institutional racism, or that they had to take these actions because it is the norm for their social environment [7,6,12,15,16,18,]. Internal motivation was identified as recognition of problem severity, often stemming from longer durations of use, theoretically leading to having more “evidence” of a drug problem [15,16]. These internal and external psychological factors led to lower or higher levels of motivation, with increased negative influences yielding lower motivation and insight levels.

### Measures

Half of the articles (n=8) utilized the commonly used Texas Christian University Criminal Justice Client Evaluation of Self in Treatment (CJ CEST), a series of scales that included scales to measure the predictor variable, levels of criminal thinking, motivation, engagement, and treatment participation, among other factors. Criminal thinking was the term used to describe insight, as higher levels of criminal thinking were associated with lower levels of problem awareness. The factors of criminal thinking were: entitlement; justification; power orientation; cold-heartedness; criminal rationalization; and personal irresponsibility.

Some studies did not measure motivation or any aspects of insight (n=8), but half of the studies (n=8) employed various types of measures, often multiple, aimed at assessing client characteristics such as motivation for treatment, psychological factors that could lower treatment engagement, such as criminal rationalization or not thinking drugs are a problem, or diagnoses such as Post Traumatic Stress Disorder. Sacks, et al. [6] conceptualized insight as client recognition of problem and taking responsibility for both being in treatment and succeeding in treatment. The study employed the CJ CEST as well as an additional measure, which measured client’s perception of problem severity and readiness for treatment. Sacks, et al. [6] findings differed from studies using only the CJ CEST in that participants were identified for level of understanding of problem, rather than the CJ CEST, which tests for lack of awareness. Melnick et al. [7] included the Circumstance, Motivation, and Readiness Scale. This scale attempted to measure both contextual factors affecting motivation, as well as individual motivation.

The eight articles that evaluated aspects of motivation and insight sought to actively define these terms and operationalize their effect on treatment and outcome using explicit definitions for insight and motivations including: motivation to complete treatment; commitment to change and attendance; and program participation, completion, and engagement.

### Insight and Motivation

Explicit definitions of insight were: recognition of problem; level of criminal thinking, with lower levels indicating poorer insight; and acknowledgement of problem severity. Optimal treatment outcomes were defined as: lowered rates of use and recidivism post-treatment; treatment completion; and seeking aftercare upon release from prison/court-ordered treatment. Four of the articles discussed insight and motivation in implicit, rather than explicit terms.

Studies found that insight played an important role at the program level. At the program level, insight was defined as enabling participants to be aware of problem severity and impact on current circumstances [7,13,15,18]. One goal of treatment in these studies was to increase participant awareness through clinical interventions. These authors found that such interventions had positive outcomes for participants both during and after treatment. In addition to interventions aimed at increasing insight, insight was also found to be a by-product of effective treatment. Bui and Morash [2] and Shaffer, et al. [22] both found that increased insight was a by-product of the engagement and participation processes of treatment.

Treatments that sought to improve insight and motivation were found to be most successful in reducing overall recidivism and relapse rates for offenders. All 16 studies reported, either explicitly, or implicitly, that successful treatment includes psychological changes through increased problem awareness and commitment to change, and that these changes in turn produced increased engagement, adherence, and led to lower rates of recidivism and relapse. Garner et al. [10] was the only study, however, to present a conceptual model of how treatments can increase insight and motivation, and how these interventions could improve treatment outcome.

### Outcome

Seven of the 16 studies contained statistics on recidivism rates. The studies that did not report outcome statistics theorized about how interventions strengthening participant insight and motivation will improve outcomes for participants. Of the four studies that reported outcome statistics, only Cosden et al. [15] reported findings that treatments tailored to increase insight and motivation improved outcomes. The other three studies to report outcome statistics did not explicitly describe program-level interventions, only individual-level insight and motivation as factors in outcome. None of the studies measured qualitative aspects of outcomes, such as increased wellbeing, psychological functioning, employment, etc.

Both individual insight and motivation were found to be key factors in individual outcomes for offenders in drug treatment. These factors played pathway roles in allowing the participant to engage in treatment to benefit both during and after treatment. The studies that used assessments of insight and motivation (Table 1) delivered these measures at the beginning of treatment. The studies that followed participants through treatment and recorded outcome statistics of recidivism and relapse found that participants who had higher levels of insight and motivation at the start of treatment showed lower rates of recidivism and relapse [2,16,18,22].

Studies employing the CJ CEST found that insight and motivation at the outset of treatment formed a pathway to improved engagement, adherence and participation, leading to reduced recidivism and drug use after treatment. Kubiak, Hiller, et al. [13,18] found that higher levels of individual insight and motivation during court-ordered treatment or incarceration led individuals be more likely to seek aftercare, which further decreased recidivism and relapse rates. All of these findings point to individual insight and motivation playing an important role not just during treatment, but also after treatment is completed. Studies also indicated that individual insight and motivation were sometimes more important than which treatment the participant received in determining positive outcomes for offenders [22].

## Discussion

This study sought to examine the relationship between insight and motivation, and that the presence of these factors affect treatment adherence, engagement, participation, and relapse and recidivism for offenders in drug treatment programs. It was found that both individual levels of insight and motivation, as well as treatments geared to increase levels of these factors lead to better outcomes for participants. Outcomes include seeking aftercare upon release from prison, or the court system, reduced drug use, and, most importantly, reduced recidivism rates. Specifically, as represented in Table 2, 12 studies measured insight (10 quantitatively), 10 studies measured motivation, and seven measured outcomes. However, several studies failed to measure both insight and motivation, or one of these factors and outcome. These findings are important for several reasons. In fact, only Cosden, et al. [15] measured all three factors.

Qualitative findings demonstrated that factors of insight and motivation are much more complex than current measurements account for. Table 2 shows the thematic codes found in the sample articles. The most important finding is that both insight and motivation are conceptually more complex than accounted for in available instruments. All studies mentioned that participants had levels of recognition of problem severity and motivation for treatment independent of the treatment itself, or the program level. While a particular treatment might be aimed at motivating clients to stop using substances, all of the studies mentioned that offenders enter treatment with less motivation and insight than non-offenders. Roberts, et al. [16] addressed this issue specifically, stating that treatment needs to be adapted to meet the needs of this unique population. All of the studies measuring relapse and recidivism rates pointed to emotional and family support being integral to positive outcomes, yet this topic are not addressed in measurements of insight or motivation. Specifically, family support could lead to increased motivation, but we cannot know this definitively from current research.

## Strengths

This study included several strengths. The first was the study’s purpose. Substance abuse researchers agree that insight and motivation are important factors in offenders adhering to and completing treatment [8,18,23,24]. However, this study found that few studies measure these factors, and even fewer report outcomes post-treatment or release. The findings demonstrated that definitions and conceptualizations are often unclear as to how individual and program-constructed insight and motivation affect relapse and recidivism rates for offenders. This fact meant that research in this area is underdeveloped. The findings that insight and motivation play a key role in treatment outcomes for this population indicate that the preliminary findings of this content analysis are important to inform the development and/or improvement of practice, policy, and research in this area. Knowing the state of the current literature allows researchers and clinicians to know which areas need to be strengthened and adapted.

Strength was the methodology used to analyse the sample. The methods used in this study allowed the authors to understand quantitative and qualitative aspects of the sample. From this understanding, a comprehensive picture of the issues emerged. Quantitative data yielded results that too few studies measure factors of insight, motivation, and outcome. Other findings showed that the qualitative studies sampled offered a conceptual understanding of insight and motivation beyond questionnaires. For instance, Bui and Morash [2] found that positive relationships with family during imprisonment allowed participants to gain better understanding of problem severity. These individuals were more likely to stay clean upon release. This type of qualitative insight points to the need to adapt measures of insight to include that gained from external relationships. Inductive analysis allowed for such subtle results to be hit upon.

## Limitations

There were several limitations to the studies that composed the content analysis sample of articles. The first limitation was that of study location. All of the studies used participants in programs in the South, Southwest, and California. This geographic sample limits the generalizability of the findings, despite the use of large samples in eight of the 16 studies (n ≥ 200). The nine of the 16 studies also included heterogeneous samples in terms of race/ethnicity, but gender statistics were not reported consistently, nor was age, further limiting generalizability. Also, overall lack of clear conceptualization and operationalization of the terms insight and motivation and conceptualization as to how they affect treatment and outcome made coding more difficult.

Another limitation was the fact that article access was limited to a single university’s collection. This was due to the fact that both authors were from one university. Had authors been from different universities, the sample size might have been larger. In addition, the sample was limited to full text articles. As stated above, this was due to the need to read know which studies used measurements, and to read descriptions of these measures. This factor limited the sample size further. The greatest limitation was the use of a single coder when analysing the data. This practice can lead to increased error in the findings through coding bias.

A final limitation was the fact that only one author served as conducted the search and coded the sample. While the second author assisted in creating coding themes and topics, the firth author conducted the analysis. While this factor was due to time limitations, using two authors to search for and code the sample could have yielded a larger sample and perhaps additional insight.

## Practice Implications

This study’s practice implications are important, as they directly affect offenders. These findings suggest several important practice implications. The results of the content analysis indicate that both individual level insight and motivation and treatment that foster these psychological factors improve relapse and recidivism rates for offenders. Findings suggest that drug treatment programs for offenders must be tailored to meet the unique needs of this population. The studies using the CJ CEST were able to determine that the unique psychological factors inherent in this population include levels of criminal thinking, which act as a barrier to insight, and in turn motivation. The studies indicated that though the sample included only offenders, these participants often came from treatment programs including clients without criminal histories. This finding suggests that programs treating offenders need to incorporate special interventions to address criminal thinking and its barriers to progress in treatment.

Findings coming from measurements used at the beginning of treatment, as well as the studies measuring patient attributes and recidivism rates point to the need to make insight and motivation assessments at the start of treatment standard practice, in order to identify individual deficits in commitment to treatment that could contribute to relapse or recidivism post-treatment. In addition, several studies found that individuals with higher motivation to succeed in treatment were able to identify the need to improve social networks and coping mechanisms aimed at sustaining abstinence from use or moderation of usage after treatment. These findings indicate that motivation and insight play a key role in behaviours during and after treatment beyond simple adherence to program and not engaging in criminal activity after treatment ends. Offenders in drug treatment need interventions aimed at increasing their awareness of the need for support and coping skills, and enabling them to create these recovery-promoting structures.

There are some issues with current practices for offenders in substance abuse treatment. Data does not exist to show what percentage of criminally involved individuals with substance abuse issues is given treatment. Nor are the measures mentioned in this study used widely in treatment. In addition, given the fact that offenders are often mandated for treatment, research is limited on how current treatment models should be adapted to better serve this population [5,9,15,9]. Because factors of insight and motivation are not actively measured, specifically not in relation to outcome, it is as yet unclear how exactly these factors affect outcomes.

## Policy Implications

Though this study focused more explicitly on practice issues, the findings suggest several implications for policy. The sample found that individual level and program-constructed insight and motivation play a role in treatment outcomes of recidivism and relapse rates for offenders. Several of the studies also identified participant-use of support networks and behaviour coping skills learned in treatment as important factors contributing to the outcome pathway just described. Yet several of the studies found that treatment for offenders with drug abuse problems is not the norm for prisoners or those involved in the court system [2,7,15,13,22]. Cosden et al. [15] in particular found that the use of treatments meant to increase participant motivation reduced recidivism in offenders with histories of drug abuse. These findings indicate the need for policies aimed at ensuring that all offenders needing drug treatment are identified and treated, in order to reduce recidivism and drug-related crime. Policies aimed at ensuring individualized treatment for offenders is also needed, as Knight et al., Garner et al. and Sacks, et al. in particular identified that individualized treatment improves outcome for this population, specifically [6,10,12,].

## Future Directions for Research

There are several topics needing to be addressed in future research. First, there need to be studies specifically aimed at conceptualizing and measuring how individual level insight and motivation affect recidivism and relapse rates for offenders in the US. Proposed studies would need to clearly define and measure these characteristics at the beginning of treatment, supply outcome statistics, and use a heterogeneous sample representative of this population. They would also need to account for sociodemographic variables that could affect insight and motivation. Studies need to be done that identify which aspects of treatment strengthen these factors in participants, and whether specific models of treatment currently in use are more useful in effecting these changes in participants and their outcomes.

## Conclusion

This content analysis was useful for several reasons. The most important finding was that while authors agreed on the importance of assessing individual insight and motivation in relation to outcomes of relapse and recidivism rates, only one study successfully did this. In addition, qualitative findings were that insight and motivation are more complex than the levels at which current instruments assess these factors. Increased recidivism in this population without proper treatment means a burden on the already strained prison system, as well as on the families and loved ones of these individuals. Relapse for those with drug abuse issues can mean overdose and death, or increased usage and consequences over time, as Cosden et al. [15] reported. The consequences of ineffective treatment or treatment that is not tailored to the specific needs of this population are dire. This study allowed for detailed understanding of the current state of the evidence, the gaps in research, as well as directions for future practice, policy, and research.

## Figures and Tables

**Table 1:** Use and Types of Measurements in Sample.

Study Authors (Qual./Quant, N)	Presence of Measure (Instrument Used)		
	Insight	Motivation	Outcome
Bui and Morash [2] (Qual, N=20)			X (RR 1Yr PR)
Carlson, et al. [23] (Qual, N=12)	X (attitude toward change)		
Cosden et al. [15] (Quant, N=801))	X (Addiction Severity Index)	X (Addiction Severity Index)	X (RR 1Yr PR)
Garner et al. [10] (Quant, N=3,266)	X (TCU Client Evaluation of Self in Treatment- externalization as block)	X (TCU Client Evaluation of Self)	
Hiller, et al. [18] (Quant, N= 419)	X (TCU Resident Evaluation of Self in Treatment- externalization as block)	X (TCU Resident Evaluation of Self)	
Knight et al. [12] (Quant, N= 3,266)	X (TCU Client Evaluation of Self in Treatment- externalization as block)	X (TCU Client Evaluation of Self in Treatment)	
Kras [3] (Qual, N= 36)	X (awareness of problem severity)		
Kubiak [13] (Quant, N= 199)	X (PTSD as block to insight)		X (RR 1Yr PR)
Magyar, et al. [8] (Quant, N= 331)	X (Personality Assessment Inventory as block to insight)		
Mattson, et al. [17] (Quant, N= 133)	X (Minnesota Multiphasic Personality Inventory-2 Restructured Form as block to insight)	X (Minnesota Multiphasic Personality Inventory-2 Restructured Form as block to motivation)	X (completion of treatment)
McKendrick, et al. [13] (Quant, N= 139)	X (Antisocial Personality Disorder diagnosis as block to insight)	X (Antisocial Personality Disorder diagnosis as block to motivation)	X (RR 1Yr PR)
Melnick et al. [7] (Quant, N= 110)		X (Client Motivation and Readiness Scale	X (RR 1Yr PR)
Pelissiersand Jones [24] (Quant, N= 1,489)		X (Motivation Assessment Scale)	
Roberts et al. [16] (Quant, N= 3,266)	X (TCU Client Evaluation of Self in Treatment- externalization as block)	X (TCU Client Evaluation of Self in Treatment)	
Sacks, et al. [6] (Quant, N= 1,170)		X (Client Assessment Inventory)	
Shaffer et al. [22] (Quant, N= 171)	X (reporting substance abuse problem)		X (RR 1Yr PR)

* Notes: Qual./Quant.= Qualitative/quantitative study. RR 1Yr PR= Recidivism rate 1 year post-release (from prison or treatment)

**Table 2:** Codes and Themes Found in the Dataset

Personal Level			Program Level		
Insight	Motivation	Treatment Outcome	Insight	Motivation	Treatment Outcome
Before versus during treatment	This population has lower motivation	Higher levels lead to better treatment outcome	Treatment improves insight	Operationalized as during- treatment participation/ engagement (motivation as action)	Treatment that focuses on increasing motivation/ insight leads to less recidivism/relapse
This population has lower insight	Internal versus external	Higher levels of motivation/insight lead to seeking aftercare	Insight as goal of treatment	Pathway process of treatment affecting individual’s behaviour	Motivation is more important to outcome than problem severity
Operationalized as criminal thinking levels/awareness of severity of problem/ awareness of role in current circumstances	Operationalized as readiness to change	Higher levels of motivation/insight lead to less recidivism	Adaptation of insight into identity (leads to behaviour change)	Increasing client participation increases personal motivation	Treatment that increases insight allows participants to make better after-treatment decisions
Longer duration of use leads to increased awareness of problem	Affects behaviour during treatment		Lack of insight leads to increased resistance to treatment/progress	Increasing client motivation increases willpower to reduce relapse rate	
Increased insight leads to behaviour change	Longer duration of use leads to higher motivation		Treatment uses motivation as a tool to increase participation and commitment to treatment	Treatment acts as external motivation that increases internal motivation	
Increased insight leads to behaviour change	Motivation leads to choices			Treatment uses motivation as a pathway to engagement and rapport	
Insight as a latent variable	Alone, not effective				
